# Medicare and Private Insurance Variations in New Medical Technology: The Case of Drug Eluting Stents

**Published:** 2016-05-18

**Authors:** Esra Eren Bayindir, Pinar Karaca Mandic

**Affiliations:** 1 Ipek University, Department of Economics, Ankara, Turkey; 2 University of Minnesota, School of Public Health, Division of Health Policy and Management, Minneapolis, MN, USA

**Keywords:** Geographic variations, Hospital variations, Medical technology diffusion

## Abstract

**Importance:**

Little is known about the geographic and hospital variations of the new medical technologies in Medicare. Even less is known about these variations for the privately insured.

**Objective:**

To examine geographic and hospital variations in the diffusion of drug eluting stents, comparing Medicare and privately insured populations.

**Design:**

Retrospective analyses of discharges from the State Inpatient Databases for 11 states (2004–2005) supplemented with data on hospital characteristics from the American Hospital Association Annual Survey.

**Setting/participants:**

Study sample included discharges with percutaneous coronary intervention (PCI) procedures that involved a cardiac stent.

**Exposure:**

Insurance type: Medicare versus private insurance.

**Main outcome:**

Use of a drug eluting stent during the PCI was our outcome variable. We estimated linear probability models at the discharge level that related our outcome variable to patient and hospital characteristics separately for Medicare and private insurance. To examine variations across hospital referral regions (HRRs) and across hospitals, our models included HRR and hospital indicators respectively.

**Results:**

Our analysis included 390,649 records (237,991 Medicare, 152,658 private insurance). We found large HRR variations in the use of drug eluting stents in 2004 for both payer types, the year after drug eluting stents were approved (adjusted CoV: 0.35 (Medicare); 0.24 (Private Insurance)). We also found large hospital variations in 2004 (adjusted CoV: 0.32 (Medicare); 0.29 (Private Insurance)). Between 2004 and 2005, adjusted HRR and hospital variations decreased across both payer types, suggesting that practice styles converged as the drug eluting stents diffused and became more common. Finally, adjusted drug eluting stent rates were highly correlated both at the HRR and hospital level across payer types.

**Conclusion:**

Our findings are consistent with the hypothesis that private insurance closely follows the lead of Medicare in terms of medical technology coverage and reimbursement.

## Introduction

A large body of research documents significant variation in the timing, intensity, and appropriateness of the use and spending of medical care across geographic regions in Medicare population [1–11]. This variation suggests that there may be important under-utilization and/or over-utilization of medical care and that there is an opportunity to improve efficiency in health care.

Little is known about the geographic and hospital variations of the new medical technologies in Medicare. Recently [12] identified important variations in drug eluting stents across hospitals for Medicare. Even less is known about these variations for the privately insured. Similar factors that drive observed Medicare geographic variations in various medical services can also result in variations in new medical technology use. Furthermore, even within the same hospital, patients may have differential access to new medical technologies based on their insurance type and generosity, intensifying the variations across payer types [13,14]. These variations could imply that the gains from medical technology are not optimally distributed across patient populations. Understanding whether and how such variations differ between Medicare and privately insured populations is important for designing payment policies and insurance benefits that encourage diffusion of high value medical technology, if needed, by tailoring policies by payer types.

Several studies compared geographic variations in Medicare to other payer types focusing on the use of inpatient discharges, days, resource use and end-of-life care, finding strong positive correlations for Medicare and privately insured across hospital referral regions (HRRs) and Core Based Statistical Areas (CBSAs) [15–19]. In contrast, corresponding spending variations were only modestly correlated, primarily attributed to pricing and reimbursement differences as private insurers negotiate differently with local providers [17,20]. Other recent evidence by Baker et al. [18] suggests that while Medicare and private insurance prices are highly correlated, the relationship between private insurance prices and Medicare volume explain the modest spending correlation. Spending is the product of prices and quantity of services, and in cases where private patients are less profitable, hospitals substitute away their resource allocations from privately insured patients to Medicare patients weakening the spending correlations.

In this paper, we focus on geographic and hospital variations in the diffusion of new technologies, with a focus on drug eluting stents, comparing Medicare and privately insured populations. Conceptually, innate differences in the preferences and market environments of cardiologists and hospitals could translate into differences in how they perceive the benefits and costs of new technologies. Physicians are differentiated with their knowledge, productivity and their reputation [21]. For example, a physician might value a reputation for practicing technologically savvy medicine. In recent work, Mandic et al. [22], showed several cardiologist characteristics to be related to drug eluting stent adoption. For example, male cardiologists, cardiologists in urban areas, and those with higher percutaneous coronary intervention (PCI) volume were faster adopters of drug eluting stents. Similarly, the same study identified several hospitals characteristics associated with adoption of drug eluting stents. For example higher volume, teaching and cardiac intensive care hospitals adopted drug eluting stents fasters.

Market environment, such as competition with other physicians over patients could also influence physician decision to use new technologies. Mandic et al. [22], showed that cardiologists in more competitive markets adopted drug eluting stents faster in the Medicare population. Overall, these physicians, hospital and market level factors are expected to contribute to hospital and geographic variations in medical technology diffusion for all payer types.

Drug eluting stents, approved by the Food and Drug Administration (FDA) April 2003, were considered a path breaking technology over the existing bare metal stents as studies found they were associated with reduced restenosis rates. Even before the FDA approval of drug eluting stents, Centers for Medicare and Medicaid Services (CMS) announced in August 2002 that Medicare would reimburse these newer stents more generously than the bare metal stents [23]. Drug eluting stents were placed in a new ambulatory payment classification (APC) in the outpatient setting, and in a new Diagnostic Related Group (DRG) in the inpatient setting, both of which received a more generous reimbursement to offset the additional cost of acquiring the drug eluting stents. Physician reimbursement by CMS, on the other hand, did not vary by the type of stent.

We examined how and whether the diffusion of drug eluting stents varied between Medicare and private insurance across hospitals and HRRs among patients who received a coronary stent between 2004 and 2005. We used national data from the State Inpatient Databases for 11 states supplemented with data on hospital characteristics from the American Hospital Association Annual Surveys. The drug eluting stent technology can be viewed as one that improves quality of cardiovascular procedures without changing reimbursements to the physicians, and one that largely compensates hospitals with its associated additional costs. As such, examining use of drug eluting stents when they first became available offers an opportunity to understand the extent to which hospital and geographic variations in technology diffusion exist, and are driven primarily by physician and hospital preferences towards improving quality of care.

## Methods

### Data

We used data from the State Inpatient Databases (SID) for 11 states: Arizona, Arkansas, Florida, Iowa, Maryland, Massachusetts, New Jersey, New York, Rhode Island, Washington and Wisconsin. The SID includes all of the patient discharge abstracts in participating states and provided as part of the Healthcare Cost and Utilization Project (HCUP). Because drug eluting stents were approved for use mid-2003, we used data from 2004 and 2005 to study their use in inpatient settings. We merged SID with data from the American Hospital Association to construct measures of hospital characteristics.

### Study sample

We included discharges by Medicare or privately insured patients age 18 or older who had a percutaneous coronary intervention (PCI) procedure that involved the use of least one cardiac stent. Final study sample consisted of 390,649 inpatient records from 11 states, 80 HRRs, and 451 hospitals.

### Measures

Our main outcome variable was a binary indicator of whether a drug eluting stent was used in the procedure (identified by ICD-9-CM codes 0055, 3607 and DRG codes 526,527). We controlled for a rich set of patient characteristics including age groups (18–44, 45–64, 65–74 and 75-up), gender, race (white, black, Hispanic, Asian and other race or missing), Charlson index of comorbidity [24–26], source of admission (emergency department, another hospital, other health facility, routine and missing), type of admission (emergency, urgent and elective), admission quarter of the patient and whether the patient was admitted at the weekend.

In analyses of variations across HRRs, we also controlled for numerous hospital characteristics. These included teaching status, service types (general hospital, heart hospital and other specialty hospital), ownership type (not-for-profit, for-profit and government), number of beds, number of nurses per bed and whether the hospital was a heart transplant hospital, shaped beam radiation system hospital, intensity-modulated radiation therapy hospital, electron beam or multislice computed tomography hospital, diagnostic/invasive catheterization hospital, interventional cardiac catheterization hospital, cardiac surgery hospital, cardiac intensive care hospital, magnetic resonance imaging hospital, positron emission or single photon emission tomography hospital, whether the hospital had an emergency department, primary care department, urgent care center and conducted health screenings.

### Statistical analyses

To examine variations across HRRs, we estimated linear probability models at the admission level that predicted the use of a drug eluting stent as a function of patient characteristics, hospital characteristics, indicators for the quarters of admission, and indicators for the HRRs. We estimated three models (unadjusted, adjusted for patient characteristics and adjusted for patient and hospital characteristics) separately for Medicare and Private Insurance. In each model, we excluded HRRs with fewer than 20 admissions of PCI procedures that involved a cardiac stent per quarter each for Medicare and private insurance patients. The parameter estimates on HRR indicators (HRR fixed effects) reflected variations across HRRs after controlling for observed factors listed above. Using the parameter estimates on HRR indicators and setting all other covariates at their mean values, we estimated adjusted rates of drug eluting stents. We computed the coefficient of variation of the unadjusted and adjusted drug eluting stent rates by HRR and compared them across payer types for each year. We also examined the correlations of unadjusted and adjusted drug eluting stent rates by payer type for each year.

To investigate variations in drug eluting stent use across hospitals, we estimated linear probability models at the admission level controlling for the same patient characteristics. Because hospitals do not cross HRRs, these analyses included indicators for hospitals (hospital fixed effects) instead of indicators for HRRs. We excluded hospitals with fewer than 20 admissions of PCI procedures that involved a cardiac stent per quarter each for Medicare and private insurance patients. We estimated unadjusted and adjusted rates of drug eluting stents by hospital and year for Medicare and Private Insurance. Similarly, we compared unadjusted and adjusted coefficient of variation (CoV) in drug eluting stent rates between Medicare and private insurance by year. Analyses were conducted using State 12 (College Station, TX).

## Results

Our analysis included a total of 390,649 inpatient records for PCIs with a cardiac stent representing 237,991 paid by Medicare and 152,658 by private insurance. In 2004, privately insured patients had higher unadjusted drug-eluting stent rate (80.54%) than Medicare (72.04%). From 2004 to 2005, drug-eluting stent rates increased for both payer types and reached 88.44% for privately insured patients and 80.90% for Medicare patients (Table 1).

Age and gender distribution of the study populations varied substantially between Medicare and privately insured (Table 1). While 42% of Medicare patients were female, only 24% of privately insured patients were female. Not surprisingly, almost 90% of the Medicare patients were 65 years or older. On the other hand, 79.66% of the privately insured patients were between ages 45 and 64. More than 70% of the Medicare and privately insured patients were White. Procedure and hospital characteristics of the study populations were largely similar across Medicare and privately insured patients (Table 1).

The distribution of unadjusted and adjusted rates of drug eluting stent use at the HRR level by payer type is reported in Table 2. Interestingly, both in 2004 and 2005, unadjusted variations (column I) for both payer types were similar to the variations adjusted for patient characteristics (column II), but largely smaller than those adjusted for patient and hospital characteristics (column III). For example, in 2004, the interquartile ranges for unadjusted variations were smaller than the corresponding range in the fully adjusted models (Medicare: 0.67 to 0.78 in column I, and 0.53 to 0.74 in column III; Private Insurance: 0.76 to 0.85 in column I, and 0.65 to 0.83 in column III). Similarly, unadjusted CoV were smaller than the adjusted CoV for both payer types, in both 2004 and 2005.

In 2004, while the unadjusted CoV (column I) were similar for Medicare and the privately insured patients, CoV adjusted for patient and hospital characteristics (column III) were smaller for privately insured patients (Medicare: 0.35; Private Insurance: 0.24). Between 2004 and 2005, dispersion of the adjusted drug eluting stent rates decreased for both payer types, with a much larger decrease for Medicare patients, and the adjusted CoV was about the same in 2015 for the two payer types (Medicare: 0.18; Private Insurance: 0.17).

Table 3 reports the distribution of unadjusted and adjusted rates of drug eluting stent use at the hospital level by payer type. In comparison with the unadjusted variations across HRRs reported in Table 2, unadjusted variations across hospitals were larger for both payer types in both years. For example unadjusted CoV across hospitals in 2004 (column I) were 0.32 and 0.29 for Medicare and private insurance respectively. In contrast, corresponding CoV across HRRs were 0.14 and 0.12 (Table 2). Table 3 also shows that CoV across hospitals adjusted for patient characteristics (column II) were the same as corresponding unadjusted CoV.

In 2004, hospital variations were similar for Medicare and privately insured patients. For example the adjusted CoV (column II) were 0.32 and 0.29 for Medicare and private insurance respectively. Similar to the case of HRRs, unadjusted and adjusted variations across hospitals also decreased from 2004 to 2005 across both payer types, although the reductions were not as large as those observed in the case of variations across HRRs.

Table 4 reports the correlations of unadjusted and adjusted drug eluting stent use by payer type at hospital referral region and hospital level separately for 2004 and 2005. In 2004, the correlation of unadjusted drug eluting stent rate (column I) between Medicare-Privately insured was high (0.95 at the HRR level, 0.97 at the hospital level). Corresponding correlation of adjusted drug eluting stent rates (column II) were also high (0.96 at the HRR and hospital levels).

From 2004 to 2005, correlation of unadjusted drug eluting stent rates (column I) at the HRR level declined (0.95 in 2004, 0.79 in 2005) Correlations adjusted for patient characteristics at the HRR level (column II) also declined (0.96 in 2004, 0.80 in 2005), whereas correlations adjusted for patient and hospital characteristics (column III) largely remained stable (0.81 in 2004, 0.84 in 2005). At the hospital level, both the unadjusted and the adjusted correlations decreased from 2004 to 2005.

## Discussion

Consistent with prior research in Medicare that showed large variations across HRRs in medical care use, we found large variations in the use of drug eluting stents in 2004, the year after drug eluting stents were approved. Interestingly, unadjusted variations were similar to those adjusted for patient characteristics alone, but substantially smaller than those adjusted for both the patient and hospital characteristics. The same pattern of larger adjusted HRR variations relative to unadjusted HRR variations held also for the privately insured patients. These findings suggest that drug eluting stent use varied by hospital characteristics, and the composition of hospitals varied by HRRs. Omission of the hospital characteristics in predicting drug eluting stent use underestimates the differences across HRRs.

The unadjusted HRR variations for privately insured patients were similar in magnitude to those for the Medicare patients, but corresponding adjusted variations were smaller in magnitude than those for the Medicare patients. This latter finding suggests differences in hospital characteristics, local hospital market characteristics as well as practice patterns of physicians affiliated with the hospitals treating Medicare and private insurance patients are important determinants of these variations. In particular, this finding provides suggestive evidence that there was greater patient and provider heterogeneity in the privately insured population that was related to the use of drug eluting stents. Another interpretation of this finding points to the large reimbursement variations for drug eluting stents among the privately insured patients. We were not able to directly control for drug eluting stent reimbursement differences across insurance plans, physicians and hospitals for the privately insured patients that could cause some of these heterogeneities. To the extent that patient and hospitals characteristics we included capture some of these differences, adjusted HRR variations would be expected to be lower for the privately insured in comparison with Medicare. We were also not able to directly control for physician level factors due to lack of data on physician identifiers in some states. Controlling for physician characteristics would likely further reduce the adjusted HRR variations in both the Medicare and privately insured populations.

Between 2004 and 2005, adjusted HRR variations decreased across both payer types. Medicare patients, who had a larger HRR variation in 2004, experienced a larger decline. By 2005, adjusted HRR variations were about the same for Medicare and privately insured patients, suggesting that practice styles converged as the drug eluting stents diffused and became more common, and the extent of convergence was larger for Medicare patients. It could also be that privately insured patients selected similar hospitals in 2004 and 2005 even though these hospitals differed in practice styles and local markets.

We also found large variations in drug eluting stent rates across hospitals for both payer types. Unadjusted variations were only slightly smaller than adjusted variations suggesting that patient characteristics that varied across hospitals did not largely influence drug eluting stent use. In 2004, adjusted hospital variations were similar for Medicare and privately insured patients. As in the case of HRRs, adjusted variations across hospitals decreased from 2004 to 2005 for both payer types, and remained similar for Medicare and private insurance, suggesting that practice styles of physicians converged as drug eluting stents became more mainstream technology. While we were not able to directly control for physician characteristics and practice styles contributing to the use of drug eluting stents, our findings are consistent with other studies that provide evidence those physicians vary in their tendency to adopt new technology and that earlier adopters are different than later adopters (Mandic et al. [22]). As the technology becomes more main stream in the later periods, physician heterogeneity contributing to hospital variations in technology use is expected to diminish.

Finally, in 2004, both the HRR level and hospital level drug eluting stent rates in private insurance were highly positively correlated with those in Medicare suggesting that similar market characteristics and practice patterns are important determinants of early technology diffusion for these payer types. In 2005, adjusted drug eluting stent rate correlations between Medicare and privately insured remained largely stable across HRRs, but declined across hospitals. This finding suggests that Medicare and privately insured patients varied more in 2005 in the types of hospitals where they received their PCIs as well as in the types of physicians that performed their PCIs.

Our study had several limitations. First, we used state inpatient databases of 11 states rather than all U.S. states. These states were selected from a subset of states that had data on hospital identifiers enabling the merge to the AHA data on hospital characteristics. They are geographically diverse, and represent about 28% of the U.S. population. Second, we did not observe information on health plan type, and plan generosity. Benefit design features may be particularly important for private insurance patients as a large body of literature shows patients respond to higher out-of-pocket payments. Third, our analytic approach dropped HRRs and hospitals with fewer than 20 cardiac stent implantations per quarter for each payer type. Variations could be different in low volume HRRs and hospitals than those we reported on in this study. Fourth, physician characteristics, which are expected to be related to the use of drug eluting stents, were not included in the analyses due to lack of data on physician identifiers in some states. Finally, drug eluting stents may not be appropriate for all patients. For example, drug-eluting stents require dual antiplatelet therapy for at least a year, and for life if tolerated, because stopping antiplatelet therapy can lead to a higher rate of thrombosis. While it would have been desirable to identify patients who are less likely to tolerate or adhere to antiplatelet therapy such as those with high risk of bleeding, discharge data are limited in doing so.

Despite its limitations, our study provides the first analysis of geographic and hospital variations of a new medical technology by Medicare and privately insured patients. As a key take-away point, we found similar adjusted variations in the use of drug eluting stents across both payer types during the first year following the new technology which generally declined during the second year. This finding confirms that, as with other medical services, different practice styles influence early diffusion of medical technologies, and as the technology becomes more common, practice styles converge. Another important finding from our study was the high correlation in variations between Medicare and private insurance consistently across HRRs and across hospitals both during the year following medical technology introduction and afterwards. This finding is consistent with the hypothesis that private insurance closely follows the lead of Medicare in terms of medical treatment coverage and reimbursement decisions.

## Figures and Tables

**Table 1:** Selected Descriptive Statistics of percutaneous coronary interventions with a cardiac stent (2004, 2005) by payer type.

	Medicare	Privately Insured	p values for unequal variance t-test
Mean drug-eluting stent rate			
2004 2005	72.04 (44.88)	80.54 (39.59)	<0.001
	80.90 (39.31)	88.44 (31.97)	<0.001
Patient and Procedure Characteristics			
% Female	41.67 (49.30)	23.82 (42.60)	<0.001
% Age 18–44	0.71 (8.38)	8.16 (27.37)	<0.001
% Age 45–64	10.04 (30.05)	79.66 (40.25)	<0.001
% Age 65–74	43.99 (49.64)	9.40 (29.18)	<0.001
% Age 75 and older	45.26 (49.78)	2.79 (16.46)	<0.001
% African American	5.94 (23.64)	5.51 (22.81)	<0.001
% White	75.45 (43.04)	72.35 (44.73)	<0.001
% Asian	0.67 (8.13)	1.18 (10.79)	<0.001
% Other Race	13.30 (33.96)	16.69 (37.28)	<0.001
% Hispanic	4.64 (21.03)	4.28 (20.24)	<0.001
Charlson score, Mean	1.22 (1.12)	0.95 (0.92)	<0.001
% Emergency procedure	32.60 (46.87)	36.10 (48.03)	<0.001
% Urgent procedure	29.30 (45.51)	29.77 (45.73)	0.002
% Elective procedure	38.06 (48.55)	34.08 (47.40)	<0.001
Hospital Characteristics			
% Teaching hospital	61.94 (48.55)	65.36 (47.58)	<0.001
% Not-for-profit	81.19 (39.08)	84.38 (36.31)	<0.001
% For-profit	11.34 (31.70)	8.11 (27.30)	<0.001
% Government	7.47 (26.29)	7.51 (26.36)	0.6336
Mean number of beds	501.33 (382.49)	512.07 (384.38)	<0.001
% General hospital	98.29 (12.97)	98.17 (13.41)	0.0056
% Heart hospital	1.50 (12.15)	1.58 (12.48)	0.0366
% Other specialty hospital	0.21 (4.63)	0.25 (5.00)	0.0257
%Cardiac surgery hospital	82.71 (37.82)	83.19 (37.39)	<0.001
% Cardiac intensive care hospital	87.81 (32.71)	88.10 (32.38)	0.0069
Number of observations	237,991	152,658	

**Source:** Authors’ analysis of the 2004–2005 State Inpatient Databases (SID) for 11 states.

**Note:** Standard deviations are reported in parenthesis.

**Table 2:** Variations in unadjusted and adjusted drug eluting stent rate by payer type, across hospital referral regions for 2004 and 2005.

Medicare				Privately Insured		
.	I	II	III	I	II	III
Mean (SD)						
2004	0.72 (0.10)	0.68 (0.11)	0.66 (0.23)	0.79 (0.9)	0.76 (0.10)	0.75 (0.18)
2005	0.78 (0.13)	0.79 (0.13)	0.80 (0.15)	0.86 (0.12)	0.87 (0.11)	0.87 (0.15)
25^th^ Percentile						
2004	0.67	0.62	0.53	0.76	0.71	0.65
2005	0.76	0.78	0.73	0.86	0.86	0.83
50^th^ Percentile						
2004	0.73	0.7	0.65	0.8	0.78	0.73
2005	0.81	0.81	0.83	0.89	0.89	0.88
75^th^ Percentile						
2004	0.78	0.76	0.74	0.85	0.82	0.83
2005	0.86	0.86	0.88	0.91	0.91	0.94
Coefficient of Variation						
2004	0.14	0.17	0.35	0.12	0.14	0.24
2005	0.17	0.16	0.18	0.14	0.13	0.17
Number of HRRs						
2004	71					
2005	71					

**Source:** Authors’ analysis of the 2004–2005 State Inpatient Databases (SID) for 11 states.

**Note:** Estimates regarding unadjusted rates of drug eluting stent rate, adjusted for patient characteristics and adjusted for patient and hospital characteristics are reported in columns with the label I, II and III respectively. Adjusted rates were estimated based on linear probability models at the admission level that predicted the use of a drug eluting stent as a function of patient characteristics, hospital characteristics, indicators for the quarters of admission, and indicators for the HRRs. Models were estimated separately by payer types. HRRs with fewer than 20 admissions of PCI procedures that involved a cardiac stent per quarter each for Medicare and private insurance patients were excluded. The parameter estimates on HRR indicators reflected variations across HRRs after controlling for observed factors listed above. Using the parameter estimates on HRR indicators and setting all other covariates at their mean values, we estimated adjusted rates of drug eluting stents by HRR.

**Table 3:** Variations in unadjusted and adjusted drug eluting stent rate by payer type, across hospitals for 2004 and 2005.

	Medicare		Privately Insured	
	I	II	I	II
Mean (SD)				
2004	0.67 (0.22)	0.64 (0.20)	0.74 (0.22)	0.71 (0.20)
2005	0.77 (0.19)	0.78 (0.18)	0.85 (0.15)	0.86 (0.15)
25^th^ Percentile				
2004	0.63	0.56	0.72	0.67
2005	0.76	0.75	0.84	0.84
50^th^ Percentile				
2004	0.73	0.69	0.81	0.77
2005	0.82	0.82	0.9	0.9
75^th^ Percentile				
2004	0.81	0.76	0.86	0.83
2005	0.88	0.89	0.93	0.93
Coefficient of Variation				
2004	0.32	0.32	0.29	0.29
2005	0.24	0.24	0.18	0.18
Number of hospitals				
2004	185			
2005	185			

**Source:** Authors’ analysis of the 2004–2005 State Inpatient Databases (SID) for 11 states.

**Note:** Estimates regarding unadjusted rates of drug eluting stent rate, adjusted for patient characteristics are reported in columns with the label I and II respectively. Adjusted rates were estimated based on linear probability models at the admission level that predicted the use of a drug eluting stent as a function of patient characteristics, indicators for the quarters of admission, and indicators for the hospitals. Models were estimated separately by payer types. Hospitals with fewer than 20 admissions of PCI procedures that involved a cardiac stent per quarter each for Medicare and private insurance patients were excluded. The parameter estimates on hospital indicators reflected variations across hospitals after controlling for observed factors listed above. Using the parameter estimates on hospital indicators, and setting all other covariates at their mean values, we estimated adjusted rates of drug eluting stents by hospital.

**Table 4:** Correlations of unadjusted and adjusted drug eluting stent use by payer type (Medicare and privately insured)

HRR level, 2004			HRR level, 2005		
I	II	III	I	II	III
0.95	0.96	0.81	0.79	0.8	0.84
**Hospital level, 2004**			**Hospital level, 200**5		
I	II		I	II	
0.97	0.96		0.91	0.92	

**Source:** Authors’ analysis of the 2004–2005 State Inpatient Databases (SID) for 11 states.

**Note:** Estimates regarding unadjusted rates of drug eluting stent rate, adjusted for patient characteristics and adjusted for patient and hospital characteristics by HRRs and by hospitals are reported in columns with the label I, II and III respectively.
